# Non-coding RNAs in polycystic ovary syndrome: a systematic review and meta-analysis

**DOI:** 10.1186/s12958-020-00687-9

**Published:** 2021-01-14

**Authors:** Liangshan Mu, Xiaoting Sun, Mixue Tu, Dan Zhang

**Affiliations:** 1 Key Laboratory of Reproductive Genetics (Ministry of Education) and Department of Reproductive Endocrinology, Women’s Hospital, Zhejiang University School of Medicine, Hangzhou, Zhejiang 310006 People’s Republic of China; 2 Wenzhou Medical University, Wenzhou, Zhejiang 325000 People’s Republic of China

**Keywords:** Polycystic ovary syndrome, Non-coding RNA, microRNA, Long non-coding RNA, circRNA

## Abstract

**Background:**

Genetic, environmental and epigenetical factors may play important roles in the pathogenesis of polycystic ovary syndrome (PCOS), however the etiology of PCOS remains unclear. Studies indicated that non-coding RNAs (ncRNAs) were involved in the occurrence and development of PCOS. Thus, we aim to perform a systematic review and meta-analysis to investigate the presence and dysregulated expression of ncRNAs in human PCOS.

**Methods:**

We searched in PubMed, Medline, Web of Science and Embase until July 2019 and summarized all eligible publications focusing on microRNAs (miRNAs), long non-coding RNAs (lncRNAs), circular RNAs (circRNAs) and small interfering RNAs (siRNAs) in PCOS.

**Results:**

Sixty-seven articles were included in our systematic review and 9 articles were included in meta-analysis. There is little overlap between studies when comparing miRNA profiles. Sensitivity analysis showed that the expression of miR-93 was upregulated in PCOS patients (WMD 0.75, *P* < 0.00001), without heterogeneity among remaining studies (I^2^ = 0%).

**Conclusion:**

A large number of ncRNAs with altered levels were observed in plasma, serum, follicular fluid, granulosa cells or other issues from PCOS patients. Aberrant ncRNAs expression in PCOS may lead to aberrant steroidogenesis, adipocyte dysfunction, altered ovarian cell proliferation and/or apoptosis and have the potential to be used as diagnostic biomarkers.

**Supplementary Information:**

The online version contains supplementary material available at 10.1186/s12958-020-00687-9.

## Background

Polycystic ovary syndrome (PCOS) is a common, multifactorial endocrine and metabolic disorder that have been estimated to affect approximately 5 to 20% of reproductive-aged women [1, 2]. All existing diagnostic criteria for PCOS are based on endocrinological and reproductive characteristics, including polycystic ovarian morphology, anovulation and clinical and/or biochemical hyperandrogenism with the exclusion of other adrenal, pituitary or androgenic disorders [3]. In addition to association with infertility and increased risk of pregnancy complications, a considerable portion of patients with PCOS are also characterized by obesity, hirsutism, insulin resistance (IR) and increased risk of type 2 diabetes mellitus (T2DM), dyslipidemia, hypertension, cardiovascular disease and endometrial cancer [4, 5]. Although some studies suggested that genetic, environmental and epigenetical factors may play important roles in the pathogenesis of PCOS [6], the etiology of PCOS remains unclear.

Non-coding RNAs (ncRNAs) are functional RNAs which are not coded for protein production but serve important regulatory role in numerous biological processes [7]. According to the nucleotides length threshold of 200 nucleotides, ncRNAs are classified into small ncRNAs (sncRNAs) and long ncRNAs (lncRNAs) [8, 9]. SncRNAs were categorized into different subgroups, including microRNAs (miRNAs), small nuclear RNAs (snRNAs), small nucleolar RNAs (snoRNAs), small interfering RNAs (siRNAs), and PIMI-interacting RNAs (piRNAs) [10–12]. Circular RNAs (circRNAs), a subgroup of ncRNAs, are named for the covalently closed circular structures which do not have a canonical 5′ cap and 3′-terminal poly A tail [13]. Ribosomal RNAs (rRNAs) and transfer RNAs (tRNAs) are also well-known ncRNAs which are highly abundant and serve key functions in RNA translation. NcRNAs regulate expression of target genes at post-transcriptional level which facilitate the occurrence and development of diseases [14]. Recently, some researches indicated that ncRNAs were involved in occurrence and development of PCOS. There were significantly differential expressions of sncRNAs in serum, granulosa cells (GCs), follicular fluid (FF) and other tissues between PCOS patients and PCOS-free population [15, 16]. Therefore, analysis of the differential expression of ncRNAs in patients with PCOS may have the possibility of be used as diagnostic biomarkers and therapeutic targets [17].

The aim of this systematic review and meta-analysis was to investigate the presence and dysregulated expression of ncRNAs in human PCOS and discuss the potential roles of these different types of ncRNAs in the pathophysiology of PCOS. Understanding the underlying molecular mechanisms of this syndrome may help to improve effective diagnosis and treatment.

## Materials and methods

Our systematic literature search adhered to the standard criteria Preferred Reporting Items for Systematic Reviews and Meta-Analysis (PRISMA) [18, 19].

### Search strategy

A comprehensive literature search was conducted in various electronic databases, including PubMed, Medline, Web of Science and Embase. Each of the terms “non-coding RNA”, “noncoding RNA”, “ncRNA”, “miRNA”, “microRNA”, “siRNA”, “snoRNA”, “circRNA” and “lncRNA” was combined (using “AND”) with each of the following terms “Polycystic ovary syndrome”, “PCOS”, “stein-leventhal” as well as with “ovary sclerocystic” and “ovary degeneration”. All articles published before July 2019 were considered for eligibility.

### Eligible studies and data extraction

Eligible studies had to meet the following criteria: 1) case–control or cohort studies; 2) original articles evaluated the expression of ncRNAs between PCOS patients and health controls; 3) reported mean expression level and fold changes (FC) of included ncRNAs. Reviews, abstracts, animal models, articles not in English or looking at drugs regulating ncRNAs in PCOS were excluded.

From eligible articles presenting original data, we extracted the information including name of the first author, year of publication, type of the samples detected, number of patients with PCOS and controls, age, body mass index (BMI) and dysregulated ncRNAs identified in PCOS.

### Quality assessment

Included studies were assessed by the Newcastle–Ottawa Scale (NOS) [20]. This scale evaluated three categories, including Selection (case definition, representativeness, control selection and control definition), Comparability of cases and controls, and Exposure (ascertainment of exposure, same method of ascertainment for cases and controls, and non-response rate). For each item within the Selection and Exposure categories, a study can be given a maximum of one star, while Comparability can be awarded a maximum of two stars. Getting five or more stars is considered a high-quality study [21].

### Statistical analysis

Among studies reporting relative FC of miRNA expression, a meta-analysis was established. Outcomes were reported as weighted mean differences (WMD). A random effect model was applied regardless of heterogeneity. The heterogeneity was calculated with both the Cochran’s Q statistic test and the I^2^ test. I^2^ > 50% indicated significant heterogeneity. If there was significant heterogeneity, we looked for potential sources of heterogeneity. For example, the results of one study were completely beyond the scope of the others, we looked for possible reasons to explain the difference. Then excluded from that study and carried out a sensitivity analysis. A subgroup analysis based on the type of sample source was also performed. All analyses were performed using the Review Manager 5.4.

## Results

### Search results

**Fig. 1:**
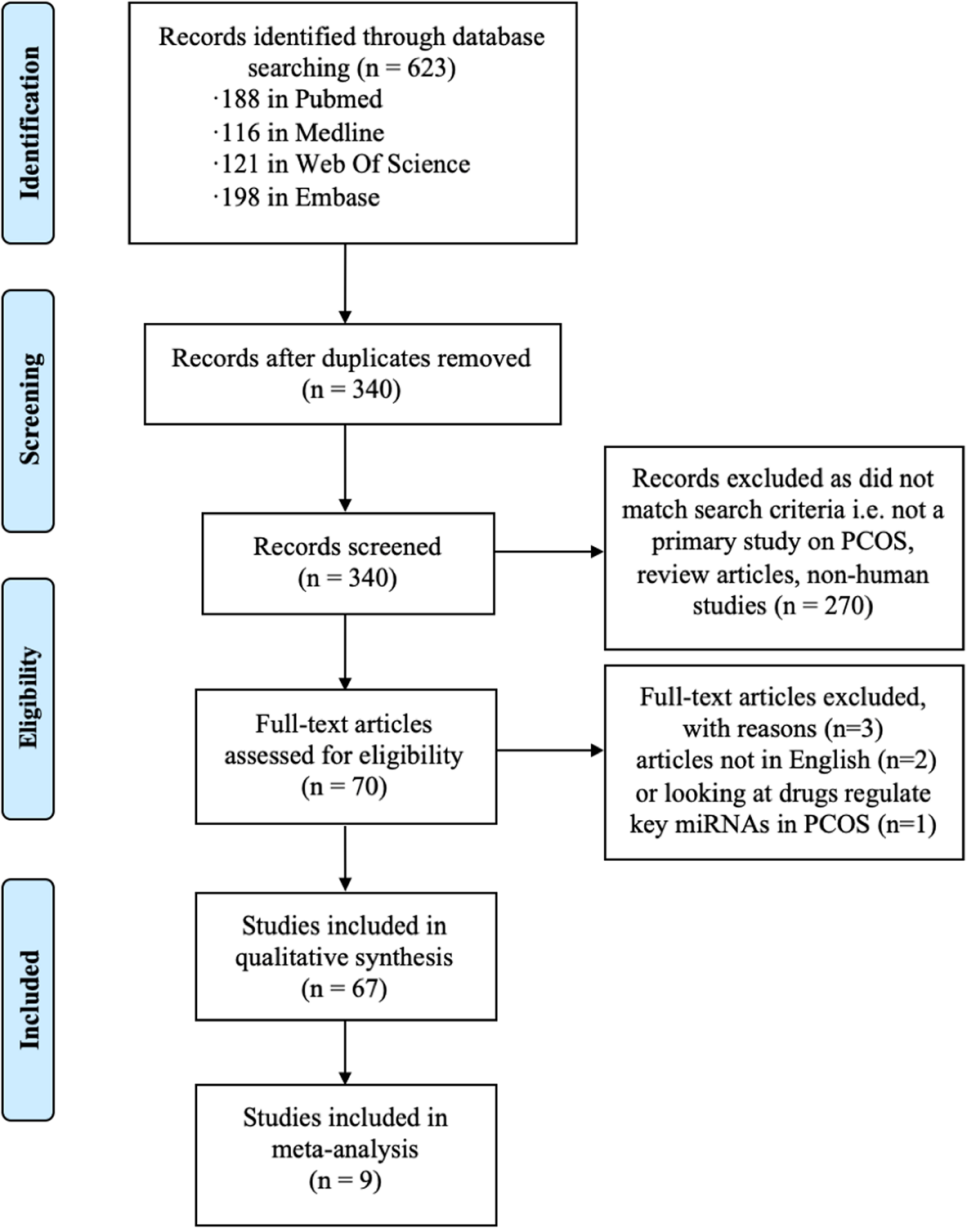
PRISMA flow methodology for selection of relevant studies. Abbreviations: PCOS, polycystic ovary syndrome

In total, we retrieved 623 articles. After exclusion of 283 multiplicate, 340 different records remained. Then, after screening based on the abstract or title, 270 studies were excluded for the following reasons: non-primary study on PCOS, reviews, abstracts, non-human articles. Next, full texts of the 70 remaining articles were examined for eligibility. Two studies were excluded for non-English publication. One study focusing on the drugs regulating miRNAs to relieve the symptoms of PCOS was also excluded. Finally, we included 67 articles in this review. A flow diagram of this literature systematic search process is presented in Fig. 1.

The details for the risk of bias for case-control studies are shown in Supplemental Table 1. All included studies scored 6 or more stars on the modified NOS.

We summarized all relevant published literature focusing on miRNAs, long ncRNAs (lncRNAs), circular RNAs and small interfering RNAs (siRNAs) in PCOS from human research. The results generally show limited concordance among these studies, which reflected the heterogeneity of those studies in specimen collection, selection of patients and controls, bioinformatic analysis and normalization of methods.

### MicroRNAs

MicroRNAs (miRNAs) are endogenous, small (composed of 20–24 nucleotides), single-stranded RNAs that were first identified in 1993 by Lee and colleagues [22]. MiRNAs regulate post-transcriptional gene expression through degradation of target messenger RNAs (mRNAs) or translational repression [11]. They negatively regulate expression of target genes by binding with the 3′-untranslated region (3′-UTRs) of corresponding mRNAs [10]. MiRNAs are involved in numerous important cellular processes, including cell proliferation, differentiation, apoptosis, tumorigenesis and development [22, 23].

Multiple studies have shown that expression of miRNAs were altered in serum, whole blood, adipose tissue, GCs, FF, and other reproductive tissues between PCOS patients and PCOS-free population [24–27]. In patients with PCOS, differentially expressed miRNAs are involved in a variety of signal pathways, including amino acid metabolism, hormone regulation, cell differentiation, and so on [13, 28]. In consequence, abnormal expression of miRNAs may provide new insight into investigating mechanisms of the pathogenesis of PCOS and exploring the potential biomarkers of disease progression.

#### Serum/plasma miRNAs in PCOS

MicroRNAs are stable, resistant to ribozyme activity and easily to be detected in serum. Therefore, miRNAs can serve as potential non-invasive biomarkers for PCOS [29]. Seventeen studies have shown that miRNAs are altered in whole blood, serum or plasma samples in women with and without PCOS [15, 24, 30–44] (Table 1). The miRNAs identified showed little consistency between these studies, which are probably due to the considerable heterogeneity of specimen collection, experimental design, bioinformatic assessment of the studies and standardized methods.

**Table 1:** Studies evaluating altered miRNA expression in women with and without PCOS

Study	Detected in tissue/cell	No. of PCOS and control	Age of PCOS/ control (years)	BMI of PCOS/ control (m/kg2)	Dysregulated miRNAs
Murri et al. 2013 [15]	Whole blood	12 PCOS vs 12 healthy controls woman vs 12 healthy men	Lean: 27 ± 4/29 ± 3 Obese: 27 ± 2/29 ± 7	Lean: 22 ± 2/22 ± 2 Obese: 39 ± 9/27 ± 6	Upregulated: miR-21,27b, 103 and 155
Long et al. 2014 [24]	Serum	68 PCOS vs 68 controls	26.6 ± 2.8/27.9 ± 3.4	25.9 ± 3.4/22.4 ± 2.1	Upregulated: miR-222, 146a and 30c
Liyan Jiang et al. 2015 [31]	Serum	30 PCOS vs 30 controls	27.5 ± 3.2/28.1 ± 4.1	25.8 ± 2.1/22.3 ± 1.5	Upregulated: miR21,222,16,19a,30c, 146a, 24 and 186
Ding et al. 2015 [30]	Serum	18 PCOS vs 18 controls	28.3 ± 5.6/29.1 ± 4.8	31.5 ± 4.1/22.7 ± 3.2	Upregulated: miR-5706, let-7i-3 pm, 4463, 3665 and 638 Downregulated: miR-124-3p, 128, 29a-3p and let-7c
Sathyapalan et al. 2015 [32]	Plasma	25 PCOS vs 25 controls	32.1 ± 9/32.2 ± 7.7	28.8 ± 5.4/27.1 ± 5.8	Upregulated: miR-93 and 223
Song et al. 2015 [33]	Serum	67 PCOS vs 67 controls	26.7 ± 2.7/27.6 ± 3.3	24.8 ± 3.3/21.9 ± 2.2	Downregulated: miR-592,124-3p, 128, 29-3p, 16, 106b, 19a, 24, 186, let-7c and 1228
Zhao et al. 2015 [34]	Serum	30 PCOS vs 70 controls	27.70 ± 3.44/27.93 ± 3.84	24.39 ± 3.67/21.87 ± 2.93	Upregulated: miR-146a, 30c and 191 Downregulated: miR-16, 223, 212, 451 and 92a
Song et al. 2016 [36]	Serum	21 PCOS vs 21 controls	23 ± 4/24 ± 6	21.7 ± 2.3/ 22.2 ± 2.7	Downregulated: miR-4522, 324-3p, and 6767-5p
Jiang et al. 2016 [35]	Serum	30 PCOS vs 30 controls	27.16 ± 3.56/27.98 ± 3.66	22.24 ± 3.87/20.46 ± 2.44	Upregulated: miR-122, 194, and 193b Downregulated: miR-199b-5p
Xiong et al. 2017 [39]	Serum	18 PCOS vs 30 controls	25.8 ± 4.5/25.5 ± 2.3	23.96 ± 4.44/20.99 ± 3.31	Downregulated: miR-23a and 23b
Hosseini et al. 2017 [38]	Plasma	205 PCOS vs 205 controls	31.2 ± 5.5/28.5 ± 5	26.5 ± 5/25.1 ± 4.6	Upregulated: miR-146a and 222
Eisenberg et al. 2017 [37]	Serum	18 PCOS vs 15 controls	26.9 ± 4.3/26.8 ± 4.7	29.3 ± 7.1/23.6 ± 3.3	Upregulated: miR-200b and 429
Ebrahimi et al. 2018 [40]	Whole blood	180 PCOS vs 192 controls	26.8 ± 5.5/27.0 ± 4.38	23.3 ± 3.6/22.92 ± 2.86	Upregulated: miR-146a
Naji et al. 2018 [42]	Serum, granulosa-lutein cells, follicular fluid	20 PCOS vs 21 controls	29.25 ± 0.84/28.42 ± 0.91	26.48 ± 0.85/24.58 ± 0.85	Upregulated in follicular fluid: miR-182 Downregulated in granulosa-lutein cells: miR-145 and 182
Murri et al. 2018 [41]	Serum	12 PCOS vs 11 controls	Lean: 27 ± 4/28 ± 3 Obese: 27 ± 2/31 ± 6	Lean: 22 ± 2/22 ± 2 Obese: 39 ± 9/37 ± 3	Upregulated: miR-34c-5p and 548d-3p Downregulated: miR-26a-5p, 30c-5p, 107 and 199a-3p
Nanda et al. 2019 [43]	Serum	20 PCOS vs 20 controls	28.35 ± 7.45/25.15 ± 4.12	32.16 ± 4.93/22.02 ± 2.64	Upregulated: miR-122, 194, and 193b Downregulated: miR-199b-5p
Rashad et al. 2019 [44]	Serum	60 PCOS vs 40 controls	31.95 ± 7.42/32.38 ± 7.68	33.2 ± 5.73/24.9 ± 2.48	Downregulated: miR-320
Sang et al. 2013 [25]	Follicular fluid	24 PCOS vs 24 controls	29.09 ± 0.70/30.83 ± 0.90	23.38 ± 0.59/21.95 ± 0.51	Downregulated: miR-132 and 320
Roth et al. 2014 [45]	Follicular fluid	12 PCOS vs 12 controls	33.1 ± 4.4/27.1 ± 3.6	25.6 ± 6.3/23.8 ± 2.9	Upregulated: miR-32, 34c, 135a, 18b, and 9
Yin et al. 2014 [46]	Follicular fluid, granulosa cells	19 PCOS vs 15 controls	20–40/20–40	NA/NA	Upregulated: miR-320 and miR-383
Scalici et al. 2016 [47]	Follicular fluid	30 PCOS vs 91 controls	33.1 ± 3.8/34.3 ± 5.1	25.4 ± 5.3/ 22.8 ± 3.7	Upregulated: miR-30a Downregulated: miR-140 and let-7b
Sorensen et al. 2016 [48]	Follicular fluid	49 PCOS vs 21 controls	28.1 ± 4.3/ 27.8 ± 3.8	25.7 ± 5.1/ 24.2 ± 3.8	Upregulated: miR-518f-3p Downregulated: miR-24–3p, 29a, 151–3p and 574–3p
Naji et al. 2017 [49]	Follicular fluid, granulosa cells	19 Hyper-androgenic PCOS vs 22 normo-androgenic PCOS	29 ± 0.66/ 28.89 ± 1.07	27.02 ± 0.97/25.94 ± 0.75	Upregulated in granulosa cells: miR-93 Downregulated in follicular fluid: miR-93 and 21
Xue et al. 2018 [50]	Follicular fluid	3 PCOS vs 3 controls	29, 36, 38/30, 31, 36	21.51,22.13,20.22/ 21.86,21.14, 20.94	Upregulated: miR-200a-3p, 10b-3p, 200b-3p,29c-3p, 99a-3p and 125a-5p Downregulated: miR-105-3p
Yao et al. 2018 [51]	Follicular fluid	55 PCOS vs 51 controls	28.13 ± 0.41/ 27.37 ± 0.46	23.25 ± 0.45/ 21.62 ± 0.38	Downregulated: miR-335-5p
Zhang et al. 2018 [52]	Follicular fluid	20 PCOS vs 20 controls	NA/ NA	NA/ NA	Upregulated: miR-873-5p
Linlin Jiang et al. 2015 [27]	Granulosa cells	16 PCOS vs 8 controls	29.69 ± 2.39/ 31.75 ± 4.40	24.07 ± 5.33/21.17 ± 3.06	Upregulated: miR-93, 107
Shi et al. 2015 [53]	Cumulus cells	24 PCOS vs 24 controls	28.3 ± 3.3/ 28.5 ± 3.6	21.5 ± 2.5/20.7 ± 2.1	Downregulated: miR-483–5p and 486–5p
Liu et al. 2015 [54]	Cumulus cells	10 PCOS vs 10 controls	27.4 ± 2.6/ 29.4 ± 3.0	22.0 ± 3.5/ 23.5 ± 3.2	Upregulated: miR-513a-3p, 508-3p, 513b, 514, 509-5p, 513c, 144, 510, 509-3p and 508-5p Downregulated: miR-151-3p, 720, 615-3p, 127-3p, 455-3p, 342-3p and 654-3p
Xu et al. 2015 [55]	Cumulus granulosa cells	21 PCOS vs 20 controls	28.76 ± 3.51/ 29.43 ± 3.92	24.01 ± 3.39/21.68 ± 2.99	Upregulated: miR-423-3p, 3651, 3653, 151b, 1273 g-3p, 590-5p, 3648, 7845-5p, 27a-5p, 1275, 483-3p, 7-5p, 483-5p, 10a-5p, 184, 619-5p, 513b-5p, 1307-5p, 4516, 1307-3p, 514b-5p Downregulated: miR-3529-3p, 7974, 3065-5p, 214-3p, 200a-3p, 203a, 4732-5p, 423-5p, 3184-5p, 548n, 221-3p, 149-5p, 1298-5p, 193a-3p, 365a-3p, 219a-1-3p, 550b-2-5p, 144-5p, 660-5p, 548e-3p, 652-3p, 222-3p,506-5p, 193a-5p, 210-5p, 365b-5p, 330-3p, 223-3p, 186-5p, 185-5p, 92b-3p, 199b-3p, 766-5p, 15b-3p, 339-5p, 3960, 766-3p, let-7a-3p
Study	Detected in tissue/cell	No. of PCOS and control	Age of PCOS/ control (years)	BMI of PCOS/ control (m/kg2)	Dysregulated miRNAs
Huang et al. 2016 [56]	Cumulus cells	18 PCOS vs 18 controls	32.6 ± 3.1/ 34.6 ± 2.2	21.6 ± 1.5/ 21.4 ± 1.8	Upregulated: miR-135b-5p, 152, 193a-3p, 194-5p, 196a-5p, 200b-3p, 423-3p, 454-3p, 455-5p, 4659a-3p, 509–3-5p, 509-3p, 513b-5p, 652-5p, 95, 1273e
Cai et al. 2017 [57]	Granulosa cells	25 PCOS vs 25 controls	29 ± 3.5/ 29 ± 3.5	NA/ NA	Downregulated: miR-145
Zhang et al. 2017 [58]	Cumulus cells	21 PCOS vs 12 controls	28.7 ± 2.6/ 29.4 ± 3.1	21.4 ± 3.3/22.7 ± 2.9	Downregulated: miR-320a
He et al. 2018 [59]	Granulosa cells	62 PCOS vs 61 controls	28.27 ± 3.10/ 28.71 ± 2.46	24.40 ± 3.34/21.77 ± 2.37	Downregulated: miR-141 and 200c
Mao et al. 2018 [60]	Granulosa cells	43 PCOS vs 26 controls	30.2 ± 2.8/ 31.1 ± 2.1	23.2 ± 1.7/22.1 ± 1.6	Downregulated: miR-126-5p and 29a-5p
Wang et al. 2018 [61]	Granulosa cells	21 PCOS vs 13 controls	28.67 ± 3.70/ 30.00 ± 2.77	24.69 ± 1.19/23.63 ± 1.70	Upregulated: miR-27a-3p
Zhong et al. 2018 [62]	Granulosa cells, ovarian cortex	18 PCOS vs 10 controls	NA/ NA	NA/ NA	Downregulated: miR-19b
Geng et al. 2019 [63]	Granulosa cells	15 PCOS vs 15 controls	27.23 ± 1.83/ 28.53 ± 1.85	22.17 ± 2.04/21.79 ± 2.13	Upregulated: miR-99a
Li et al. 2019 [64]	Granulosa cells	46 PCOS vs 32 controls	29.21 ± 4.78/ 29.43 ± 3.82	24.35 ± 3.32/23.12 ± 2.13	Upregulated: miR-33b and 142 Downregulated: miR-423
Luo et al. 2019 [65]	Granulosa cells	20 PCOS vs 18 controls	27 ± 3.26/ 29 ± 3.22	NA/ NA	Upregulated: miR-23a
Wang et al. 2019 [66]	Granulosa cells	24 PCOS vs 21 controls	28.708 ± 0.802/29.571 ± 0.994	25.958 ± 0.836/22.173 ± 0.878	Upregulated: miR-3188 and 3135b
Song et al. 2019 [67]	Granulosa cells	63 PCOS vs 20 controls	28.21 ± 2.78/ 27.43 ± 3.62	24.35 ± 2.12/22.12 ± 1.73	Upregulated: miR-186 and 135a
Hou et al. 2019 [68]	Granulosa cells	38 PCOS vs 35 controls	29.60 ± 0.66/ 29.66 ± 0.82	25.25 ± 0.59/22.63 ± 0.53	Upregulated: miR-3188 and 3135b
McCallie et al. 2010 [69]	Blastocysts	6 PCOS vs 10 controls	NA/ NA	NA/ NA	Downregulated: miR- let-7a, 19a, 19b, 24, 92, and 93
Chen et al. 2013 [26]	Adipose tissue	11 PCOS vs 11 controls	27.46 ± 4.07/ 32.41 ± 6.61	28.56 ± 5.6/ 22.89 ± 2.65	Upregulated: miR-93, 133 and 223
Wu et al. 2014 [70]	Adipose tissue	8 PCOS with IR vs 8 PCOS without IR vs 9 controls with IR vs 6 healthy without IR	27.75 ± 4.98/ 30.00 ± 5.57/ 33.66 ± 6.37/ 32.33 ± 5.03	31.92 ± 4.95/25.49 ± 5.56/35.78 ± 7.68/22.80 ± 1.32	Upregulated in PCOS patients and controls with IR: miR-93, and 25
Lin et al. 2015 [17]	Ovarian theca interna tissues	10 PCOS vs 8 controls	28.80 ± 3.97/ 32.00 ± 2.16	24.42 ± 4.84/20.51 ± 2.06	Downregulated: miR-19b, 92a, 92b, 141, and 200a
Xiang et al. 2016 [71]	Ovary cortex	20 PCOS vs 20 controls	27.3 ± 2.5/ 28.2 ± 3.7	26.2 ± 3.7/ 22.0 ± 2.5	Downregulated: miR-483
Yuan et al. 2017 [72]	Ovarian tissue	20 PCOS vs 20 controls	NA/ NA	NA/ NA	Downregulated: miR-320
McAllister et al. 2019 [73]	Ovarian theca cells	7 PCOS vs 7 controls	NA/ NA	NA/ NA	Upregulated: miR-100-5p, 99b-5p, 1271-5p, 409-5p, 744, 410-3p, 127-3p, 654-5p, 494-3p, 1301-3p, 502-3p, 501-3p and 1293 Downregulated: miR-125a-3p,148b-5p, 195-5p,130b-3p and 4542a-5p

*Abbreviations*: *PCOS* polycystic ovary syndrome, *BMI* body mass index, *NA* not available, *IR* insulin resistance

Long et al. [24] evaluated expression of serum miRNAs using miRNA arrays in women with PCOS (*n* = 5) and age-matched controls (*n* = 5). Eight miRNAs (miR-16, miR-19a, miR-24, miR-30c, miR-106b, miR-146a, miR-186, miR-222 and miR-320) were upregulated in serum from PCOS women, while miR-320 was downregulated in the serum from PCOS women. However, quantitative polymerase chain reaction (qPCR) in women with PCOS (*n* = 68) and without PCOS (*n* = 68) confirmed that three miRNAs (miR-30c, miR-146a and miR-222) were significantly upregulated in PCOS patients. Sensitivity and specificity analysis revealed that the combination of miR-30c, miR-146a and miR-222 was suggested as potential diagnostic markers. A larger study [38], contrasting women with (*n* = 205) and without (*n* = 205) PCOS, showed that the miR-146a rs2910164 and miR-222 rs2858060 polymorphisms are significantly associated with increased risk of PCOS. Ebrahimi et al. [40] also found that the miR-146a rs2910164 polymorphism significantly differed between PCOS (*n* = 180) and healthy controls (*n* = 192).

#### Follicular fluid (FF) miRNAs in PCOS

Ovarian FF not only serves as an important microenvironment for the development of the oocyte but also contains important regulators such as ovarian cell secretions, hormones and blood plasma components that act a vital role in oocyte maturation [15]. Ten studies suggested that expression of miRNAs in FF from PCOS women was different from that of healthy controls [16, 25, 42, 45–48, 50–52] (Table 1). There is little overlap between these studies when comparing the miRNA profiles.

Roth et al. [45] found that 29 miRNAs were differentially expressed between the PCOS group (*n* = 12) and healthy controls (*n* = 12). Among these miRNAs, the expression of 5 miRNAs (miR-9, miR-18b, miR-32, miR-34c and 135a) showed significant upregulation in FF from PCOS women. Further pathway analysis revealed possible target genes involved in steroid synthesis, carbohydrate metabolism and insulin regulation [17, 74]. These target genes identified exert functions related to PCOS phenotypes.

Sang et al. [25] identified that over 100 miRNAs were significantly differentially expressed in FF from women with PCOS (*n* = 24) and non-PCOS controls (*n* = 24) undergoing intracytoplasmic sperm injection (ICSI) treatment. They found that the expression level of miR-320 was downregulated in PCOS patients compared to the healthy controls. Furthermore, another study conducted by Yin et al. [46] using FF and GCs from women with (*n* = 19) or without (*n* = 15) PCOS suggested that the expression of miR-320 was upregulated in FF and GCs from women with PCOS compared to the controls. This is contradictory to the results of study by Sang et al. The miRNA profile of FF variability between studies could be attributed to study populations, heterogenic nature of PCOS, differences in the control groups or different methods. Thus, larger well-powered studies are needed to identify candidate miRNAs which are most relevant to PCOS.

#### Granulosa cells/ cumulus cells miRNAs in PCOS

Granulosa cells (GCs) are vital for oocyte growth and maturation. Dysfunction of GCs may contribute to abnormal folliculogenesis and unbalanced hormone production in patients with PCOS [19, 49]. Cumulus cells are a unique subset of GCs which interact with the oocyte directly and are essential for regulation of oocyte metabolism [75]. To date, 19 studies have examined miRNAs in either GCs or cumulus cells taken from women with and without PCOS [16, 27, 46, 53–68] (Table 1). Collectively, these data indicate that distinct miRNA profiles do indeed exist in GCs / cumulus cells between PCOS group and healthy controls. Potential pathogenic miRNAs play a multi-faceted role in hormone regulation, cellular proliferation and angiogenesis.

Amongst these differentially expressed miRNAs, miR-513b has showed significant upregulation in cumulus cells of PCOS patients in three studies [54–56]. In addition, upregulation of miR-423-3p in cumulus cells of PCOS women was reported by two studies [55, 56]. MiR-509-3p, a miRNA identified in Huang’s [56] and Liu’s [54], also shown up-regulated expression in cumulus cells from PCOS women.

In GCs from women with PCOS, miR-93 showed significant upregulation as reported by two studies [16, 27], while miR-145 was downregulated in another two studies compared to control group [42, 57].

#### Other tissues miRNAs in PCOS

Eight studies have revealed that expression of miRNAs was altered in other samples of PCOS population, including blastocysts [69], adipose tissue [26, 70], ovarian theca cells [73], ovarian theca interna tissues [17], ovary cortex [62, 71] and ovarian tissue [72]. McCallie et al. [69] reported that blastocysts isolated from patients with PCOS exhibited significantly decreased expression of six miRNAs (miR-let-7a, miR-19a, miR-19b, miR-24, miR-92, and miR-93) in comparison with donor fertile control blastocysts. Two studies suggested that the expression of miR-93 was significantly increased in adipose tissue from IR/non-PCOS and all PCOS patients compared with controls [26, 70]. Furthermore, expression of miR-483 was significantly decreased in ovary cortex lesion from PCOS patients. It is conjectured that miR-483 inhibited cell proliferation possibly by targeting IGF1, and may be an alternative biomarker for PCOS diagnosis and treatment.

#### Meta-analysis for miRNAs expression

Due to the limited consistency between these studies, if mean expression level and FC of same miRNA was reported in more than three articles, this miRNA was selected to perform our meta-analysis. Finally, only miR-320 and miR-93 were included. Our meta-analysis included a total of 9 individual studies, of which 5 studies reported on miR-320 and 4 studies reported on miR-93. Real time polymerase chain reaction (RT-PCR) was used to measure the expression of miRNAs in all studies. Among these studies, two detected miRNA expression in two different tissues [16, 46]. Three studies examined the expression of miRNAs in FF, three in GCs, one in Cumulus cells, one in serum, one in plasma, one in adipose tissue, and one in ovarian tissue.

**Fig. 2:**
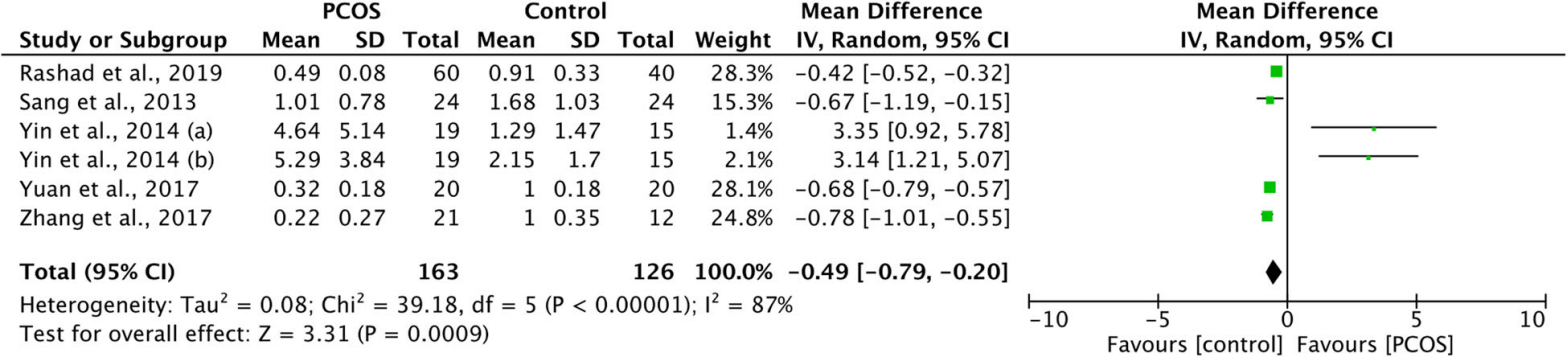
Forest plot of miR-320 expression in PCOS patients and controls. Abbreviations: PCOS, polycystic ovary syndrome

Forest plot of miR-320 expression in PCOS patients and controls is shown in Fig. 2. The meta-analysis revealed that the expression of miR-320 was downregulated in PCOS patients compared to healthy controls (WMD -0.49, 95%CI − 0.79 to − 0.20, *P* < 0.001); however, significant heterogeneity was existed among included studies (I^2^ = 87%). To identify the potential sources of heterogeneity, subgroup analysis was performed based on type of sample source. For the expression of miR-320, there was no significant difference among studies detected in FF (two trials, WMD 1.16, 95%CI − 2.77 to 5.08), studies detected in GCs (two trials, WMD 1.06, 95%CI − 2.78 to 4.98) and studies detected in other tissues by the test of interaction (*P* = 0.37, Supplemental Figure 1). The reasons for this heterogeneity have not been identified.

**Fig. 3:**
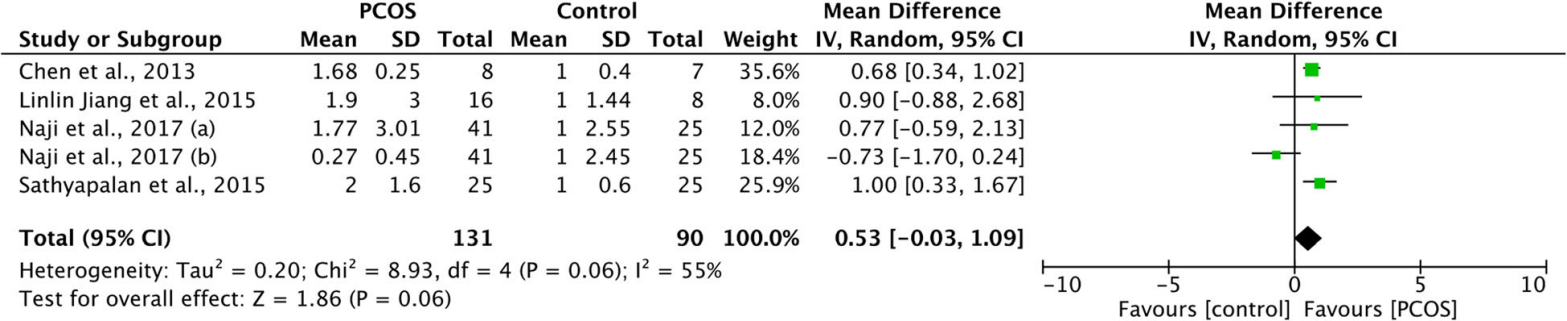
Forest plot of miR-93 expression in PCOS patients and controls. Abbreviations: PCOS, polycystic ovary syndrome

Forest plot of miR-93 expression in PCOS compared to the controls is shown in Fig. 3. The expression of miR-93 was upregulated in PCOS patients compared to healthy controls, however, the difference between two groups was not significant (WMD 0.53, 95%CI − 0.03 to 1.09, *P* = 0.06), with significant heterogeneity among included studies (I^2^ = 55%).

#### Sensitivity analysis

**Fig. 4:**
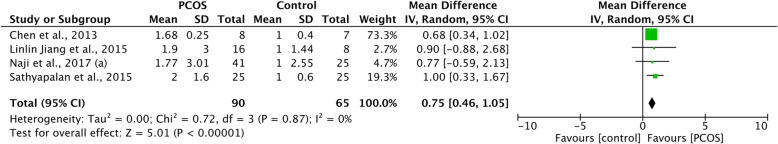
Sensitivity analysis of miR-93 expression in PCOS patients and controls. Abbreviations: PCOS, polycystic ovary syndrome

Significant heterogeneity existed among studies for the expression of miR-93 (I^2^ = 55%). The study conducted by Naji et al. [16] showed the miR-93 expression in FF was out of range of other studies and may contribute to the heterogeneity. After excluding this article, the result showed that compared with healthy controls, the expression of miR-93 was upregulated in PCOS patients (WMD 0.75, 95%CI 0.46 to 1.05, *P* < 0.00001, Fig. 4), without heterogeneity among remaining studies (I^2^ = 0%).

### Long non-coding RNAs

Long non-coding RNAs (lncRNAs) play a crucial part in cell proliferation, differentiation, apoptosis and tumorigenesis via interactions with chromatin modification, RNA-binding proteins, and ceRNA networks [76, 77]. Previous studies have shown that lncRNAs may be involved in follicle development. For example, a study found that lncRNA Neat1 knockout (KO) mice failed to establish successful pregnancy due to low serum progesterone level and corpus luteum dysfunction [78]. To date, ten studies have investigated lncRNAs in women with and without PCOS [79–88] (Table 2).

**Table 2:** Studies evaluating altered lncRNA expression in women with and without PCOS

Study	Detected in tissue/cell	No. of PCOS and control	Age of PCOS/ control (years)	BMI of PCOS/ control (m/kg2)	Dysregulated lncRNAs
Liu et al. 2015 [79]	Peripheral blood leukocytes	23 PCOS vs 17 controls	28.44 ± 0.37/29.71 ± 0.44	28.88±0.37/27.01 ± 1.1	Upregulated: lncRNA CTBP1-AS
Liu et al. 2015 [80]	Peripheral blood leukocytes	23 PCOS vs 17 controls	28.44 ± 0.37/29.71 ± 0.44	27.74 ± 1.56/26.61 ± 1.10	Upregulated: lncRNA SRA
Huang et al. 2016 [81]	Cumulus cells	35 PCOS vs 35 controls	32.6 ± 3.1/ 34.6 ± 2.2	21.6 ± 1.5/ 21.4 ± 1.8	620 upregulated and 3 downregulated The most upregulated: lncRNA ENST00000433673, The most downregulated: lncRNA NR_027436
Liu et al. 2017 [82]	Granulosa cells	7 PCOS vs 7 controls	29.71 ± 3.59/30.14 ± 2.97	20.8 ± 1.96/ 22.34 ± 1.12	692 upregulated and 170 downregulated The most upregulated: lncRNA ENST00000533082, The most downregulated: lncRNA ENST00000448179
Huang et al. 2018 [83]	Cumulus cells	30 PCOS vs 30 controls	32.6 ± 3.1/ 33.6 ± 2.2	21.6 ± 1.5/ 21.4 ± 1.6	Upregulated: lncRNA PWRN2
Jiao et al. 2018 [84]	Follicular fluid	10 PCOS vs 8 controls	30.6 ± 3.86/ 30.0 ± 4.21	25.9 ± 3.14/ 24.7 ± 3.69	A total of 1583 novel lncRNAs were identified
Lin et al. 2018 [85]	Serum	16 PCOS without IR vs 30 PCOS with IR vs 30 healthy controls	28.3 ± 3.1/ 31.3 ± 4.6/ 26.8 ± 6.7	20.9 ± 3.6/ 25.1 ± 3.5/19.5 ± 2.4	Downregulated in PCOS patients with IR: lncRNA GAS5
Zhao et al. 2018 [86]	Luteinized granulosa cells	30 PCOS vs 30 controls	28 ± 3/ 29 ± 3	23.3 ± 2.9/ 21.8 ± 2.4	Upregulated: LINC-01572:28
Yang et al. 2019 [87]	Granulosa cells	44 PCOS vs 34 controls	31.2 ± 3.4/ 30.9 ± 3.1	NA/ NA	Upregulated: lncRNA BANCR
Li et al. 2019 [88]	Peripheral blood leukocytes	20 PCOS vs 20 controls	28.45 ± 1.35/ 31.7 ± 1.52	25.21 ± 0.86/ 20.56 ± 0.58	Upregulated: lncRNA H19

*Abbreviations*: *PCOS* polycystic ovary syndrome, *BMI* body mass index, *NA* not available, *IR* insulin resistance

Three studies have examined lncRNAs in peripheral blood leukocytes of women with PCOS [79, 80, 88]. Liu et al. [79] evaluated C-Terminal binding protein 1 antisense (CTBP1-AS) in peripheral blood leukocytes in women with PCOS (*n* = 23) and healthy controls (*n* = 17). Researchers found that expression of CTBP1-AS was significantly higher in women with PCOS. Further study by Liu et al. observed that expression of lncRNA SRA was overtly elevated in PCOS patients compared with healthy women [80]. Another study by Li et al. showed expression levels of lncRNA H19 in peripheral blood leukocytes from PCOS women were significantly higher than in healthy controls. Women with higher expression levels of lncRNA H19 have a significantly higher risk of PCOS than individuals with lower expression level. Those findings indicated that elevated lncRNA H19 levels may be a useful biomarker for early stage of PCOS for susceptible individuals. However, limitations of these three studies should not be ignored, such as the relatively small sample size, and the study sample selection of peripheral blood leukocytes. As we all know, PCOS is a systemic endocrine disease, which cannot be reflected merely by peripheral blood leukocytes.

Four studies have examined lncRNAs in GCs / cumulus cells of women with PCOS [81–83, 87]. Two studies used a microarray to compare lncRNA profiles in cumulus cells / GCs between women with and without PCOS. Huang et al. [83] showed that 620 lncRNAs expressions were upregulated and only three lncRNAs expressions were down-regulated in PCOS cumulus cells, while Liu et al. [82] found that 692 lncRNAs expressions were up-regulated and 170 lncRNAs expressions were down-regulated in PCOS GCs. These data all indicate that up-regulated expression of lncRNAs in PCOS was more common than that of down-regulation.

On the whole, these studies have identified a number of lncRNAs that proved to aggravate development of PCOS. However, discrepancy between the results of different studies implies that existing evidence is not enough to draw firm conclusions so far.

### Circular RNAs

Circular RNAs (circRNAs) are generated from precursor mRNA (pre-mRNA) via head-to-tail backsplicing and function as miRNA sponges. Many studies have demonstrated that circRNAs are differentially expressed in a variety of diseases, especially in tumors [89–92]. In recent years, expression profiles of circular RNA in preimplantation embryos and human GCs during maternal aging have been extensively studied. Some studies found that circular RNAs are closely related to the development of oocyte and embryos [93, 94].

Up to now, four studies have investigated discrepancy of circRNAs between PCOS patients and healthy controls [95–98] (Table 3). Two of them used microarray analysis to compare circRNA expression profiles in cumulus cells from women with and without PCOS in order to uncover potential biological functions [95, 96]. Che et al. have shown that a total of 1032 circRNAs’ expression was significantly changed in PCOS patients, including 311 circRNAs that were up-regulated and 721 circRNAs down-regulated [95]. Four aberrantly expressed circRNAs (hsa_circ_0083952, hsa_circ_0082709, hsa_circ_0002425, and hsa_circ_0015168) showed statistical significance after Bonferroni correction. Another study by Ma et al. revealed that a total of 286 circRNAs (167 upregulated and 119 downregulated) were significantly altered in cumulus cells of PCOS patients [96]. Further analysis showed that expression levels of hsa_circ_0043533 and hsa_circ_0043532 were significantly higher in PCOS group after quantitative real-time polymerase chain reaction (qRT-PCR) validation, while expression level of hsa_circ_0097636 was significantly lower versus the non-PCOS group. They further found that circRNAs with abnormal expression level contained miRNA-binding sites. Some miRNAs were associated with PCOS indicating that circRNAs may be implicated in PCOS by interactions with PCOS-related miRNAs. However, the size of samples in these two studies was limited. A larger population is required for further investigation.

**Table 3:** Studies evaluating altered circRNA expression in women with and without PCOS

Study	Detected in tissue/cell	No. of PCOS and control	Age of PCOS/ control (years)	BMI of PCOS/ control (m/kg2)	Dysregulated circRNAs
Che et al. 2019 [95]	Cumulus cells	20 PCOS vs 20 controls	30.4 ± 2.5/29.8 ± 3.1	22.3 ± 1.4/21.8 ± 1.5	Downregulated: circRNA-0083952, 0082709, 0002425, and 0015168
Ma et al. 2019 [96]	Cumulus cells	25 PCOS vs 25 controls	29.60 ± 2.76/ 31.16 ± 3.28	24.46 ± 2.47/ 21.16 ± 2.04	Upregulated: circRNA-0043533 and 0043532 Downregulated: circRNA-0097636
Wang et al. 2019 [97]	Exosomes of follicle fluids	3 PCOS vs 3 controls	24, 25, 27/ 26, 27, 28	22.4, 26.4, 20.3/22.9, 16, 22.3	167 upregulated and 245 downregulated The top five most upregulated: circRNA-15918_GREB1L,2702_ANKH, 7788_HTT, 5762_SPHKAP, and 8717_FANCL The top five most downregulated: circRNA-5172_NBPF20, 14485_MBOAT2, 15481_ATP6V0D1, 6172_LINC-PINT, and 6485 _LYRM4
Zhang et al. 2019 [98]	Granulosa cells	15 PCOS vs 15 controls	29.20 ± 2.42/ 29.73 ± 3.39	20.82 ± 1.68/ 19.84 ± 1.00	Upregulated: circRNA-0001577 Downregulated: circRNA-0020093

*Abbreviations*: *PCOS* polycystic ovary syndrome, *BMI* body mass index, *NA* not available

A recent study [98] explored circRNAs expression profiles in GCs from PCOS women of reproductive age. They found that 4 circRNAs expressions were upregulated in PCOS group compared with healthy controls, whereas 23 circRNAs expressions were downregulated. Gene ontology analysis showed that dysregulated circRNAs were mainly involved in inflammation, proliferation, and the vascular endothelial growth factor (VEGF) signaling pathway. Moreover, a study investigated circRNAs expression in exosomes of FF obtained from women with and without PCOS [97]. They identified that 167 circRNAs expressions were upregulated and 245 circRNAs expressions were downregulated, a circRNA–microRNA interaction network was constructed based on research data. Differentially expressed circRNAs in PCOS were linked with pathways including inflammation, bacterial infection and oxidative stress.

### Short interfering RNAs

Short interfering RNAs (siRNAs), 21–25 long nucleotides, double stranded (ds) RNAs act in gene silencing through binding to the sequence of their target mRNAs [99, 100]. Three studies have demonstrated the application of siRNA-mediated gene silencing in PCOS research and have revealed potential mechanisms in PCOS [101–103] (Table 4).

**Table 4:** The utility of siRNA mediated gene silencing approaches in PCOS research

Study	Detected in tissue/cell	No. of PCOS and control	Age of PCOS/ control (years)	BMI of PCOS/ control (m/kg2)	siRNA Transfection
Anjali et al. 2015 [101]	Granulosa cells	18 PCOS vs 30 controls	NA/ NA	NA/ NA	SiRNAs target IRS-2, PI3K, Akt
Li et al. 2016 [102]	Granulosa cells	10 PCOS vs 10 controls	NA/ NA	NA/ NA	SiRNAs target heat shock protein 90B1
Song et al. 2018 [103]	Granulosa cells	25 PCOS vs 25 controls	27.77 ± 4.34/ 29.39 ± 3.37	21.24 ± 3.59/22.02 ± 3.30	SiRNA 439, 1930, and 2117 target human insulin receptor gene

*Abbreviations*: *PCOS* polycystic ovary syndrome, *BMI* body mass index, *NA* not available

Follicle stimulating hormone (FSH) stimulates the growth and differentiation of ovarian follicles. Anjali et al. [101] confirmed that FSH specifically increases the expression of IRS-2 and decreases IRS-2 mRNA degradation in human GCs. However, in GCs of PCOS women the expression of FSH-stimulated IRS-2 was defective. They further found that after IRS-2 knockdown by siRNA, the defect of FSH may cause the deceleration of follicular growth, which can lead to infertility. These results suggested IRS-2 may contribute to the development of new therapeutic strategies for PCOS patients.

Li et al. [102] demonstrated that increased heat shock protein 90B1 (HSP90B1) levels in PCOS ovarian cells positively correlate with cell survival. Knockdown of HSP90B1 with siRNA increased apoptosis and decreased ovarian cells from patients with PCOS. Thus, the altered expression profile of HSP90B1 has an important effect on the proliferation and survival of ovarian cells, suggesting a key role of HSP90B1 in PCOS pathogenesis.

Song et al. [103] suggested that insulin induced cell apoptosis, stimulated cell proliferation and decreased telomerase activity in GCs from both PCOS women and healthy controls, but insulin receptor gene (INSR) siRNAs transfection blocked the effect of insulin. Thus, insulin induced more GCs apoptosis may be involved in the pathogenesis of PCOS.

### Pathophysiological pathways impacted by differentially expressed ncRNAs

Dysregulated ncRNAs identified in PCOS were involved in various cellular and biological pathways, some of which are implicated in PCOS lesion development (Table 5).

**Table 5:** List of ncRNAs observed in PCOS

ncRNAs	Detected in cell/tissue	Expression	Target gene(s)/acting pathway	Reported function(s)	References	
miR-93	Adipose tissue	↑	GLUT4	Correlated with insulin resistance	[26]	
	Granulose cells	↑	CDKN1A	Promotes proliferation and cell cycle progression	[27]	
	Granulose cells	↑	SMAD7 and TGFBR2	Impaired follicle development	[49]	
	Plasma	↑	NGF Signalling and HGF Signalling	Correlated with insulin resistance	[32]	
miR-320	Serum	↓	Through ET-1 regulating ERK1/2 signalling pathway	Promotes cell mitosis	[44]	
	Cumulus cells	↓	RUNX2	Estrogen deficiency	[58]	
	Ovarian tissue	↓	Through IRS-1 regulating ERK1/2 signalling pathway	Inhibits insulin resistance	[72]	
	Follicular fluid	↓	RAB5B	Correlated with insulin resistance	[25]	
	Follicular fluid, granulosa cells	↑	E2F1 and SF-1	Inhibited estradiol release and cell proliferation; enhanced progesterone synthesis	[46]	
miR-9	Follicular fluid	↑	IRS2, SYT1, IL8	Mediates the effects of insulin; steroid synthesis	[45]	
miR-18b	Follicular fluid	↑	IRS2, SYT1, IL8	Mediates the effects of insulin; steroid synthesis	[45]	
miR-135a	Follicular fluid	↑	IRS2, SYT1, IL8	Mediates the effects of insulin; steroid synthesis	[45]	
	Granulosa cells	↑	ESR2	Promoted proliferation and inhibited apoptosis	[67]	
miR-186	Granulosa cells	↑	ESR2	Promoted proliferation and inhibited apoptosis	[67]	
miR-21	Serum	↑	LATS1	Promotes secondary follicle growth	[31]	
	Granulose cells	↑	SMAD7 and TGFBR2	Impaired follicle development	[49]	
miR-592	Serum	↓	LHCGR	Inhibited cell viability and cell cycle progression	[33]	
miR-92a	Ovarian theca interna tissues	↓	GATA-6 and IRS-2	Related to androgenic and insulin signaling pathways	[17]	
miR-223	Plasma	↑	PPAR, IGF-1 and angiopoietin signaling	Correlated with insulin resistance hyperandrogenism, endometrial response and ovarian follicle development	[32]	
miR-483	Ovary cortex	↓	IGF1	Inhibits cell proliferation	[71]	
miR-483-5p	Cumulus granulosa cells	↓	Notch3 and MAPK3	Related to cell proliferation and apoptosis	[55]	
	Cumulus cells	↓	IGF2	Inhibits insulin resistance	[53]	
miR-486-5p	Cumulus cells	↓	PI3K/Akt	Promote cumulus cell proliferation	[53]	
miR-509-3p	Cumulus cells	↑	MAP3K8	Improved oestradiol secretion	[56]	
miR-6767-5p	Serum	↓		Negatively associated with fasting glucose	[36]	
miR-145	Granulosa cells	↓	IRS1	Negatively Regulates Cell Proliferation	[57]	
miR-126-5p	Granulosa cells	↓	Klotho-associated signaling	Involved in apoptosis of cells	[60]	
miR-29a-5p	Granulosa cells	↓	Klotho-associated signaling	Involved in apoptosis of cells	[60]	
miR-27a-3p	Granulosa cells	↑	SMAD5	Decreased cell proliferation and promoted cell apoptosis	[61]	
miR-335-5p	Follicular fluid	↓	SGK3	Involved in granulosa cells proliferation	[51]	
miR-873-5p	Follicular fluid	↑	Heme oxygenase-1 (HO-1)	Involved in apoptosis of cells	[52]	
miR-19b	Granulosa cells	↓	IGF-1	Promotes cell proliferation	[62]	
miR-99a	Granulosa cells	↑	IGF-1	Regulates proliferation and apoptosis	[63]	
miR-33b	Granulosa cells	↑	TGFBR1	Induced dysregulated cell proliferation, apoptosis, and cell cycle	[64]	
miR-142	Granulosa cells	↑	*TGFBR1*	Induced dysregulated cell proliferation, apoptosis, and cell cycle	[64]	
miR-423	Granulosa cells	↓	SMAD7	Induced dysregulated cell proliferation, apoptosis, and cell cycle	[64]	
miR-23a	Granulosa cells	↑	SIRT1	Promotes cell apoptosis	[65]	
miR-130b-3p	Ovarian theca cells	↑	DENND1A Variant 2	Correlated with androgen biosynthesis	[73]	
lncRNA CTBP1-AS	Peripheral blood leukocytes	↑		Reguletes androgen receptor AR activity	[79]	
lncRNA SRA	Peripheral blood leukocytes	↑		Promote activity of steroid receptors	[80]	
lncRNA PWRN2	Cumulus cells	↑	miR-92b-3p and TMEM120B	Correlated with oocyte nuclear maturation	[83]	
lncRNA GAS5	Serum	↓		Correlated with insulin resistance,cell apoptosis and proliferation	[85]	
lncRNA BANCR	Granulosa cells	↑	Bax and p53	promote apoptosis	[87]	
lncRNA LINC-01572:28	Luteinized granulosa cells	↑	SKP2 and p27	inhibits cell proliferation and cell cycle	[86]	
lncRNA H19	Peripheral blood leukocytes	↑		correlated with fasting plasma glucose levels	[88]	

*Abbreviations*: *TGFBR1* transforming growth factor beta receptor 1, *NGF* nerve growth factor, *HGF* hepatic growth factor, *ET-1* endothelin-1, *IRS1* insulin receptor substrate 1, *IRS* insulin receptor substrate, *SYT1* synaptogamin 1, *IL8* interleukin 8, *LATS1* large tumor suppressor, *LHCGR* luteinizing hormone/chorionic gonadotropin receptor, *GATA6* GATA-binding factor 6, *IRS-2* insulin receptor substrate proteins 2, *PPAR* peroxisome proliferator receptor, *IGF-1* insulin like growth factor-1, *SGK3* serum/glucocorticoid-regulated kinase family member 3, *↑* the expression of ncRNAs was upregulated, *↓* the expression of ncRNAs was downregulated

#### Altered steroidogenesis

Previous research reported that miR-423-3p directly interacts with AdipoR2 (adiponectin receptor 2) [104]. The expression level of adiponectin receptor 2(AdipoR2) in theca cells of PCOS women was significantly lower than that in normal ovaries. AdipoR2 is a receptor to adiponectin. Destruction of adiponectin and/or adiponectin receptors interfere with normal progesterone production and plays an important part in pathogenesis of hyperandrogenism in PCOS [105, 106]. Downregulated miR-592 expression in PCOS patients induced a significant increase of luteinizing hormone/chorionic gonadotropin receptor (LHCGR) mRNA expression, which is also an important factor of hyperandrogenemia in PCOS.

Androgens are important for female reproduction. However, androgens cannot stimulate corresponding targets without androgen receptors, whose abnormality may lead to reproductive defects [107–109]. A study has illustrated that expression of androgen receptors was increased in women with PCOS [110]. Follicular development defects were observed in androgen receptors gene knockout model, suggesting that androgen receptors is also actively involved in sustaining normal ovarian function [111]. A study showed that extra-nuclear androgen receptor signals could enhance expression of anti-apoptotic miR-125b. Then, the increased expression of miR-125b may contribute to androgen-induced follicular survival by reducing the number of follicular atresia [112].

In addition, abnormal expression of estrogen receptors may be related to pathogenesis and abnormal follicular development in PCOS as well [113]. Targeted disruption of estrogen receptor-alpha gene in female mice exhibited high level of LH, cystic ovaries and ovulation abnormalities [114]. Moreover, studies have shown that miR-193b and miR-222 could influence estrogen receptor by targeting estrogen receptor 1 (ESR1) gene. MiR-193b has been shown to regulate estrogen signaling [113], while miRNA-222 negatively regulates estrogen receptor-alpha expression at the protein level [115]. Interestingly, miR-222 and 193b also have an influence on steroid secretion. Besides, Long et al. found miR-146a was negatively correlated with serum testosterone levels in PCOS women [24]. The study by Huang et al. [56] indicated that miR-509-3p improved the secretion of oestradiol by inhibiting expression of MAP 3 K8. These results will help to illuminate regulation of steroid secretion in the pathogenesis of PCOS.

LncRNAs, which regulate response of androgen, estrogen and progesterone receptors have been identified, suggesting that lncRNAs act a vital role in the hormone-regulatory networks. For instance, previous studies have shown that, lncRNA SRA has the ability to promote activity of steroid receptors [116–119]. Then, Liu et al. found that the expression level of lncRNA SRA in peripheral blood leukocytes was significantly upregulated in women with PCOS than that in the healthy controls [80]. Thus, there is potential correlation between lncRNA SRA and PCOS. A previous functional study [120] revealed that upregulated CTBP1-AS expression could promote expression of androgen-responsive genes and facilitate androgen receptor mediated transcriptional activity, which is consistent with findings by Liu et al. [79]. Thus, upregulated CTBP1-AS expression might have a potential in the pathogenesis of PCOS hyperandrogenism.

#### Altered insulin sensitivity and insulin resistance (IR)

Most women with PCOS have a certain degree of IR and hyperinsulinemia. Up to 70% of PCOS women have IR, and the prevalence of obesity or overweight in PCOS women is as high as 38 to 88% [4]. Studies have shown that obese patients with PCOS have a higher level of IR.

Chen et al. found that decreased expression of GLUT-4 in adipocytes is closely related to IR regardless of PCOS, and overexpressed miR-93 in adipose tissue reduced GLUT4 expression [26]. This study further observed that the expression of miR-93 was up-regulated not only in PCOS, but also in the control group with IR. Thus, miR-93 may play an important part in other IR-related diseases, such as obesity and T2DM. Wu et al. [70] also demonstrated that increased miR-93 expression in adipose tissue is correlated with PCOS pathology and IR. In another study [121], the expression level of miR-320 was elevated up to 50-fold in 3 T3-L1 adipocytes rendering IR, which was induced by treatment with high insulin and high glucose. Insulin sensitivity was restored in experiments with anti-miR-320 oligo transfection, as was evidenced by the increases of GLUT4 expression, as well as insulin-stimulated glucose uptake. Anti-miR-320 oligo is not only effective in IR adipocytes, but not in normal adipocytes. MiR-320 was found to be highly abundant in FF of women with PCOS, thus it may be a potentially target for improving IR [25].

Compared with PCOS patients without IR and non-PCOS healthy controls, serum lncRNA GAS5 was severely downregulated in PCOS women with IR [85]. The relative expression of lncRNA GAS5 in serum was negatively associated with HOMA-IR. AUC model also identified GAS5 as a good predictive biomarker for PCOS diagnosis. Taken all the data into consideration, it is shown that circulating lncRNA GAS5 may contribute to the development of IR and PCOS.

#### Altered ovarian cell proliferation and/or apoptosis

Evidence from monkey-model-based studies demonstrates that proliferation of GCs was significantly increased while the apoptosis of small antral follicles was significantly decreased with androgen treatment [122]. Furthermore, the imbalance between proliferation and apoptotic rates was also observed in women with PCOS [49]. The study showed that expression level of the proliferation marker, Ki-67, was significantly higher in the PCOS granulosa cells.

Study by Chen et al. shown that upregulated expression of miR-513b-5p promoted apoptosis and inhibited cell proliferation in gastric cancer by targeting high mobility group-box 3 protein (HMGB3) [123]. Thus, hypothesis was raised that miR-513b-5p in the cumulus cells of PCOS patients might be implicated in regulation of apoptosis, which further affected atretic process of the follicles and led to follicular maturation disorder. In women with PCOS, a study has shown that miR-320 functions as a regulator of cell proliferation and hormone synthesis through directly inhibiting the expression of E2F1 and SF-1 [46]. Overexpressed miR-93 also promotes cell proliferation by targeting CDKN1A in GCs [27]. Interestingly, higher level of miR-93 in circulation [32] and adipose tissue [26] of PCOS patients was uncovered by several studies. It is suggested that miR-93 may have a role in proliferative status of GCs and IR by targeting CDKN1A in GCs and GLUT4 in adipose tissue, respectively [27]. Decreased level of miR-145 has been demonstrated to be involved in negative regulation of GCs proliferation in PCOS by targeting insulin receptor substrate 1 (IRS1) inhibits [57].

Several lncRNAs have also been confirmed to be involved in the regulation of cell proliferation and apoptosis. A large number of studies have shown that GAS5 is implicated in cell apoptosis and proliferation [49, 124]. Decrease regulation of GAS5 in serums might play a role in the pathogenesis of PCOS. In addition, the role of lncRNA BANCR in PCOS is to promote apoptosis by upregulating pro-apoptotic p53 and Bax expression [87]. LncRNA LINC-01572:28 inhibited GC growth by decreasing p27 protein degradation in patients with PCOS [86].

## Discussion

This is the first systematic review and meta-analysis that summarized and evaluated all relevant published literature focusing on miRNAs, lncRNAs, circRNAs, and siRNAs in PCOS from human research. PCOS is a syndrome with many clinical manifestations, which has a major impact on the quality of life, especially for premenopausal women. However, it is difficult to explicitly expound the pathophysiology of PCOS, since it involves endocrinology, gynaecology, diabetology, and other areas. At present, the mechanisms underlying development of anovulation, IR and dyslipidemia in PCOS patients have not been fully clarified. Understanding molecular regulations that cause differential expression of ncRNAs is helpful to elucidate the pathogenesis of PCOS.

Our meta-analysis suggested that compared with healthy controls, the expression of miR-93 was upregulated in PCOS patients. Besides, the expression of miR-320 was downregulated in PCOS patients, with significant heterogeneity. The present systematic review suggested that a large number of ncRNAs were reported to exhibit altered levels in plasma, serum, FF, or GCs from PCOS patients compared with healthy controls. However, the miRNA profile varies between studies. This could be attributed to studied populations, the heterogenic nature of PCOS, differences in the control groups, or different methods. Thus, larger well-powered studies are needed to identify candidate miRNAs that are most relevant to PCOS. The aberrant ncRNAs expression might lead to abnormal steroidogenesis, adipocyte dysfunction, altered ovarian cell proliferation, and/or apoptosis. These ncRNA-related symptoms mentioned above help to explain the pathophysiology of PCOS. However, specific role of ncRNAs in the development of PCOS remains unclear because one ncRNA-centered pathway may have multiple mRNA targets and one mRNA 3’UTR may also be regulated by numerous ncRNAs. It is unattainable so far to distinguish whether the altered ncRNAs expression profile is the cause or the result of PCOS. However, it appears that ncRNAs could serve as effective biomarkers for PCOS diagnosis and prognosis.

The strength of this systematic review and meta-analysis is an extensive literature search. We searched main databases, including PubMed, Medline, Web of Science, and Embase. Our review outlines biological function of these PCOS-related ncRNAs from human research, which has proved that dysregulation of ncRNAs is an important factor in the pathophysiology of PCOS. There are several limitations in our study. Firstly, most of studies included in this review had a relatively small sample size, and the results were inconsistent or even contradictory. Secondly, many trials did not provide FC value, thus a limited number of studies were included in our meta-analysis.

Looking ahead, in order to make full use of ncRNAs as non-invasive diagnostic markers, large genome-wide mapping studies are still needed, including a more diverse population of study participants with good clinical characteristics. To ensure consistency of sample collection and processing protocols, it would be ideal to establish a global collaborative database of ncRNAs expression profile under PCOS and non-PCOS conditions. Moreover, due to multifactorial nature of PCOS, employing a set of ncRNAs rather than a single ncRNA as biomarkers can improve the accuracy in the diagnosis and assessment of treatment. With the advent of simple and reliable detection software combining bioinformatics analysis, clinical laboratories, and research teams are able to further assess ncRNAs expression profile in various samples. The underlying ncRNAs network in the development of PCOS will continue to be clarified in the near future.

## Conclusions

A large number of ncRNAs with altered levels were observed in plasma, serum, follicular fluid, granulosa cells or other issues from PCOS patients. The aberrant ncRNAs expression in PCOS may lead to aberrant steroidogenesis, adipocyte dysfunction, altered ovarian cell proliferation and/or apoptosis and have the potential to be used as diagnostic biomarkers.

## Supplementary Information

**Additional file 1 **: **Table S1**. Quality assessment of the included studies**.** ★, identify high quality choices with a star. All included studies scored 6 or more stars on the modified Newcastle–Ottawa Scale.

**Additional file 2 **: **Figure S1**. Subgroup analysis of miR-320 expression in PCOS patients and controls. Abbreviations: PCOS, polycystic ovary syndrome.

## Data Availability

The current study was based on the results of relevant published studies.

## Abbreviations

PCOS: Polycystic ovary syndrome
miRNAs: MicroRNAs
lncRNAs: Long non-coding RNAs
circRNAs: Circular RNAs
siRNAs: Small interfering RNAs
BMI: Body mass index
IR: Insulin resistance
FC: Fold changes
NOS: Newcastle–Ottawa Scale
WMD: Weighted mean differences
