# Supplementary material for: Non-coding RNAs in polycystic ovary syndrome: a systematic review and meta-analysis
Source: Reprod Biol Endocrinol. 2021 Jan 14;19:10. doi: 10.1186/s12958-020-00687-9 (PMC7807442; doi:10.1186/s12958-020-00687-9)
Supplement: Supplementary file 1 — Additional file 1 : Table S1. Quality assessment of the included studies. ★, identify high quality choices with a star. All included studies scored 6 or more stars on the modified Newcastle–Ottawa Scale. [file 12958_2020_687_MOESM1_ESM.docx]

**Supplemental Table 1. Quality assessment of the included studies.**

| Study | Selection |  |  |  | Comparability | Exposure |  |  | Scores |
| --- | --- | --- | --- | --- | --- | --- | --- | --- | --- |
|  | Adequate definition of cases | Representative-ness of the cases | Selection of  controls | Definition of  controls | Control for important factor | Ascertainment of exposure | Same method  to ascertain for cases and controls | Non-Response  rate | |
| MiRNAs |  |  |  |  |  |  |  |  |  |
| Murri et al., 2013 | ★ | ★ | ★ | ★ | ★★ | ★ | ★ | ★ | 9 |
| Long et al., 2014 | ★ | ★ | ★ | ★ | ★ | ★ | ★ | ★ | 8 |
| Liyan Jiang et al., 2015 | ★ | ★ | ★ | ★ | ★ | ★ | ★ | ★ | 8 |
| Ding et al., 2015 | ★ | ★ | ★ | ★ | ★ | ★ | ★ | ★ | 8 |
| Sathyapalanet  et al., 2015 | ★ | ★ | - | ★ | ★ | ★ | ★ | ★ | 7 |
| Song et al., 2015 | ★ | ★ | - | ★ | ★ | ★ | ★ | ★ | 7 |
| Zhao et al., 2015 | ★ | ★ | - | - | ★ | ★ | ★ | ★ | 6 |
| Song et al., 2016 | ★ | ★ | - | ★ | ★ | ★ | ★ | ★ | 7 |
| Jiang et al.,  2016 | ★ | ★ | ★ | ★ | ★★ | ★ | ★ | ★ | 9 |
| Study | Selection |  |  |  | Comparability | Exposure |  |  | Scores |
|  | Adequate definition of cases | Representative-ness of the cases | Selection of  controls | Definition of  controls | Control for important factor | Ascertainment of exposure | Same method  to ascertain for cases and controls | Non-Response  rate |  |
| Xiong et al., 2017 | ★ | ★ | - | ★ | ★ | ★ | ★ | ★ | 7 |
| Hosseini  et al., 2017 | ★ | ★ | - | ★ | ★ | ★ | ★ | ★ | 7 |
| Eisenberg et al., 2017 | ★ | ★ | - | ★ | ★★ | ★ | ★ | ★ | 8 |
| Ebrahimi  et al., 2018 | ★ | ★ | ★ | ★ | ★ | ★ | ★ | ★ | 8 |
| Naji et al.,  2018 | ★ | ★ | - | - | ★ | ★ | ★ | ★ | 7 |
| Murri et al.,  2018 | ★ | ★ | - | ★ | ★★ | ★ | ★ | ★ | 8 |
| Nanda et al.,  2019 | ★ | ★ | - | ★ | ★ | ★ | ★ | ★ | 7 |
| Rashad et al.,  2019 | ★ | ★ | ★ | ★ | ★★ | ★ | ★ | ★ | 9 |
| Sang et al., 2013 | ★ | ★ | - | ★ | ★ | ★ | ★ | ★ | 7 |
| Roth et al., 2014 | ★ | ★ | - | - | ★ | ★ | ★ | ★ | 6 |
| Study | Selection |  |  |  | Comparability | Exposure |  |  | Scores |
|  | Adequate definition of cases | Representative-ness of the cases | Selection of  controls | Definition of  controls | Control for important factor | Ascertainment of exposure | Same method  to ascertain for cases and controls | Non-Response  rate |  |
| Yin et al., 2014 | ★ | ★ | - | - | ★ | ★ | ★ | ★ | 6 |
| Scalici et al., 2016 | ★ | ★ | - | - | ★ | ★ | ★ | ★ | 6 |
| Sorensen et al., 2016 | ★ | ★ | - | ★ | ★★ | ★ | ★ | ★ | 8 |
| Naji et al.,  2017 | ★ | ★ | - | - | ★★ | ★ | ★ | ★ | 7 |
| Xue et al.,  2018 | ★ | ★ | - | ★ | ★★ | ★ | ★ | ★ | 8 |
| Yao et al.,  2018 | ★ | ★ | - | - | ★ | ★ | ★ | ★ | 6 |
| Zhang et al.,  2018 | ★ | ★ | - | - | ★ | ★ | ★ | ★ | 6 |
| Linlin Jiang et al., 2015 | ★ | ★ | - | - | ★ | ★ | ★ | ★ | 6 |
| Shi et al.,  2015 | ★ | ★ | - | - | ★ | ★ | ★ | ★ | 6 |
| Liu et al.,  2015 | ★ | ★ | - | ★ | ★ | ★ | ★ | ★ | 7 |
| Study | Selection |  |  |  | Comparability | Exposure |  |  | Scores |
|  | Adequate definition of cases | Representative-ness of the cases | Selection of  controls | Definition of  controls | Control for important factor | Ascertainment of exposure | Same method  to ascertain for cases and controls | Non-Response  rate |  |
| Xu et al.,  2015 | ★ | ★ | - | - | ★ | ★ | ★ | ★ | 6 |
| Huang et al.,  2016 | ★ | ★ | - | ★ | ★ | ★ | ★ | ★ | 7 |
| Cai et al.,  2017 | ★ | ★ | - | - | ★ | ★ | ★ | ★ | 6 |
| Zhang et al.,  2017 | ★ | ★ | - | - | ★ | ★ | ★ | ★ | 6 |
| He et al.,  2018 | ★ | ★ | - | - | ★ | ★ | ★ | ★ | 6 |
| Mao et al.,  2018 | ★ | ★ | - | - | ★ | ★ | ★ | ★ | 6 |
| Wang et al.,  2018 | ★ | ★ | - | - | ★ | ★ | ★ | ★ | 6 |
| Zhong et al.,  2018 | ★ | ★ | ★ | ★ | ★ | ★ | ★ | ★ | 8 |
| Geng et al.,  2019 | ★ | ★ | - | - | ★ | ★ | ★ | ★ | 6 |
| Li et al.,  2019 | ★ | ★ | - | - | ★ | ★ | ★ | ★ | 6 |
| Study | Selection |  |  |  | Comparability | Exposure |  |  | Scores |
|  | Adequate definition of cases | Representative-ness of the cases | Selection of  controls | Definition of  controls | Control for important factor | Ascertainment of exposure | Same method  to ascertain for cases and controls | Non-Response  rate |  |
| Luo et al., 2019 | ★ | ★ | - | - | ★★ | ★ | ★ | ★ | 7 |
| Wang et al.,  2019 | ★ | ★ | - | - | ★ | ★ | ★ | ★ | 6 |
| Song et al.,  2019 | ★ | ★ | - | ★ | ★ | ★ | ★ | ★ | 7 |
| Hou et al., 2019 | ★ | ★ | - | - | ★ | ★ | ★ | ★ | 6 |
| McCallie et al., 2010 | ★ | ★ | - | - | ★★ | ★ | ★ | ★ | 7 |
| Chen et al.,  2013 | ★ | ★ | - | ★ | ★★ | ★ | ★ | ★ | 8 |
| Wu et al.,  2014 | ★ | ★ | - | ★ | ★★ | ★ | ★ | ★ | 8 |
| Lin et al.,  2015 | ★ | ★ | - | ★ | ★ | ★ | ★ | ★ | 7 |
| Xiang et al.,  2016 | ★ | ★ | - | - | ★ | ★ | ★ | ★ | 6 |
| McAllister et al., 2019 | ★ | ★ | - | ★ | ★ | ★ | ★ | ★ | 7 |
| Study | Selection |  |  |  | Comparability | Exposure |  |  | Scores |
|  | Adequate definition of cases | Representative-ness of the cases | Selection of  controls | Definition of  controls | Control for important factor | Ascertainment of exposure | Same method  to ascertain for cases and controls | Non-Response  rate |  |
| Yuan et al.,  2017 | ★ | ★ | - | ★ | ★ | ★ | ★ | ★ | 7 |
| LncRNAs |  | |  |  |  |  |  | |  |
| Liu et al.,  2015 | ★ | ★ | - | - | ★ | ★ | ★ | ★ | 6 |
| Liu et al.,  2015 | ★ | ★ | - | - | ★★ | ★ | ★ | ★ | 7 |
| Huang et al.,  2016 | ★ | ★ | - | - | ★ | ★ | ★ | ★ | 6 |
| Liu et al.,  2017 | ★ | ★ | - | - | ★ | ★ | ★ | ★ | 6 |
| Huang et al.,  2018 | ★ | ★ | - | - | ★ | ★ | ★ | ★ | 6 |
| Jiao et al.,  2018 | ★ | ★ | - | - | ★ | ★ | ★ | ★ | 6 |
| Lin et al.,  2018 | ★ | ★ | - | - | ★★ | ★ | ★ | ★ | 7 |
| Zhao et al.,  2018 | ★ | ★ | - | ★ | ★ | ★ | ★ | ★ | 7 |
| Yang et al.,  2019 | ★ | ★ | - | ★ | ★ | ★ | ★ | ★ | 7 |
| Study | Selection |  |  |  | Comparability | Exposure |  |  | Scores |
|  | Adequate definition of cases | Representative-ness of the cases | Selection of  controls | Definition of  controls | Control for important factor | Ascertainment of exposure | Same method  to ascertain for cases and controls | Non-Response  rate |  |
| Li et al.,  2019 | ★ | ★ | ★ | ★ | ★ | ★ | ★ | ★ | 8 |
| CircRNAs |  | |  |  |  |  |  | |  |
| Che et al.,  2019 | ★ | ★ | - | - | ★ | ★ | ★ | ★ | 6 |
| Ma et al.,  2019 | ★ | ★ | ★ | ★ | ★ | ★ | ★ | ★ | 8 |
| Wang et al.,  2019 | ★ | ★ | - | - | ★ | ★ | ★ | ★ | 6 |
| Zhang et al.,  2019 | ★ | ★ | - | - | ★ | ★ | ★ | ★ | 6 |
| SiRNAs |  | |  |  |  |  |  |  |  |
| Anjali et al.,  2015 | ★ | ★ | - | - | ★ | ★ | ★ | ★ | 6 |
| Li et al.,  2016 | ★ | ★ | - | - | ★ | ★ | ★ | ★ | 6 |
| Song et al.,  2018 | ★ | ★ | - | ★ | ★ | ★ | ★ | ★ | 6 |
|  |  |  |  |  |  |  |  |  |  |
