# Supplementary figures and images for: Non-coding RNAs in polycystic ovary syndrome: a systematic review and meta-analysis
Source: Reprod Biol Endocrinol. 2021 Jan 14;19:10. doi: 10.1186/s12958-020-00687-9 (PMC7807442; doi:10.1186/s12958-020-00687-9)

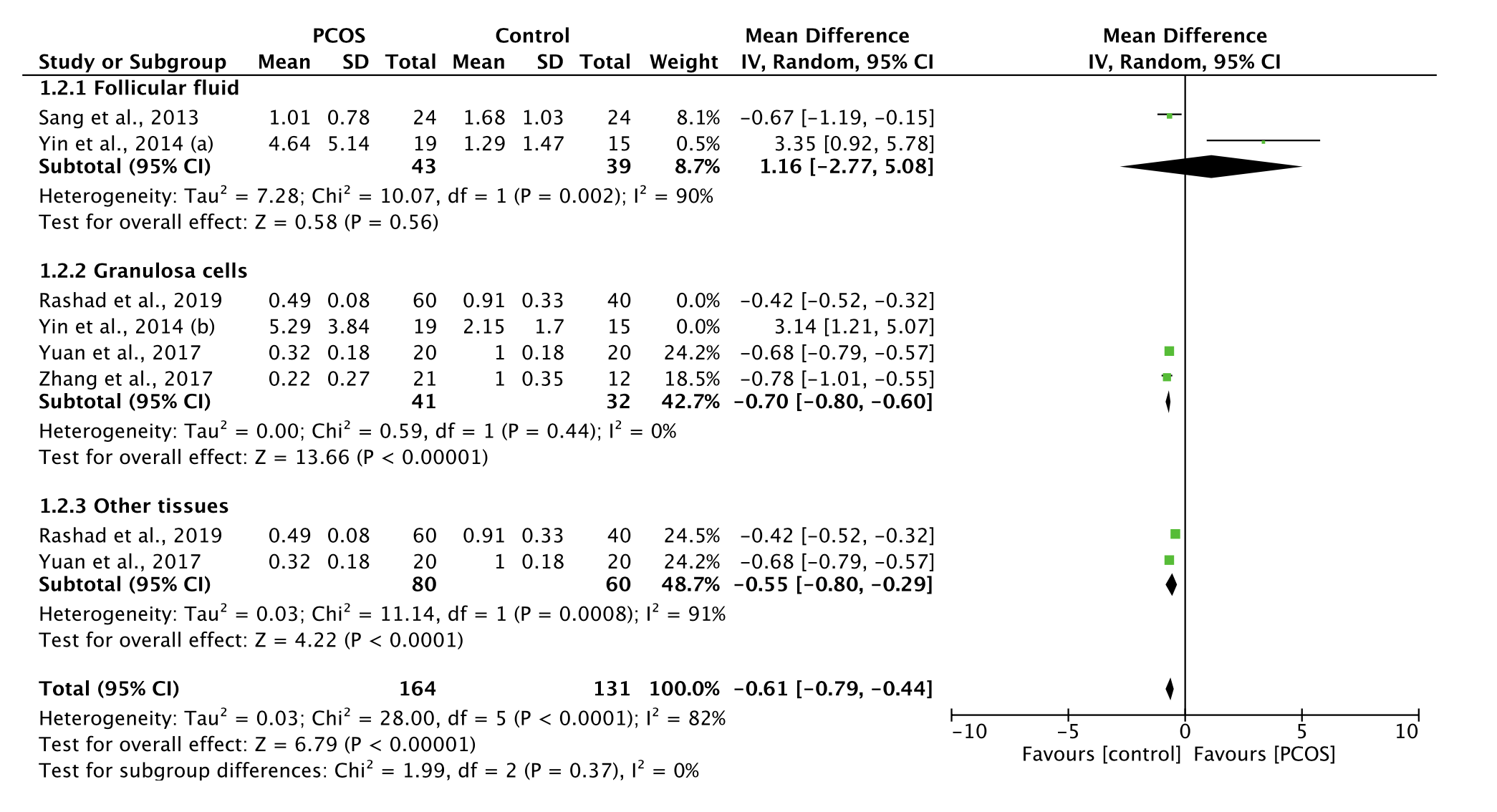

Supplement: Supplementary file 2 — Additional file 2 : Figure S1. Subgroup analysis of miR-320 expression in PCOS patients and controls. Abbreviations: PCOS, polycystic ovary syndrome. [file 12958_2020_687_MOESM2_ESM.tiff]
